# Perception towards preeclampsia and perceived barriers to early health-seeking among pregnant women in selected Hospitals of South Gondar Zone, Northwest Ethiopia: A qualitative study

**DOI:** 10.1371/journal.pone.0271502

**Published:** 2022-08-04

**Authors:** Maru Mekie, Minale Bezie, Abenezer Melkie, Dagne Addisu, Ermias Sisay Chanie, Wubet Alebachew Bayih, Shimeles Biru, Mekonnen Hailie, Tigist Seid, Enyew Dagnew, Tewachew Muche, Eshetie Molla Alemu

**Affiliations:** 1 Department of Midwifery, Debre Tabor University, College of Health Sciences, Debre Tabor, Ethiopia; 2 Department of Pediatrics and Child Health Nursing, Debre Tabor University, College of Health Sciences, Debre Tabor, Ethiopia; 3 Department of Public Health, Debre Tabor University, College of Health Sciences, Debre Tabor, Ethiopia; National Research Centre of Egypt, EGYPT

## Abstract

**Background:**

Preeclampsia is one of the top maternal morbidity and mortality that disproportionately affects pregnant women in low and middle-income countries where access and quality of health services are limited. People in different areas perceive preeclampsia differently which directly or indirectly affects the timing and place of heath seeking. Positive perception about perceived causes, perceived complications, and prevention of preeclampsia is central for the prediction and early diagnosis of the disease. However, little is known about the perception of pregnant women towards preeclampsia in Ethiopia. This study aimed to assess the perception towards preeclampsia and perceived barriers to early health-seeking among pregnant women in selected Hospitals of South Gondar Zone, Northwest Ethiopia.

**Methods:**

A qualitative study using phenomenological approach was implemented among 20 purposively selected pregnant women who visited health facilities for antenatal care service in four selected Hospitals of the South Gondar Zone of the Amhara Region. Data were collected through an in-depth interview (IDI) using a semi-structured interview guide from January to February 2020. Thematic analysis was executed using Open Code Software version 4.03.

**Results:**

The majority of the participants believed preeclampsia as a pregnancy-specific hypertensive disease and mainly associated it with overweight and nutritional problems. With regards to the perceived severity, the study participants agreed that preeclampsia can lead women to death. Personal delay, lack of awareness about the disease, transport problem, and low socioeconomic condition were perceived as the major reasons for the delay to early health-seeking (the 1^st^ and the 2^nd^ delay). While poor service provision and long waiting times were the barriers to receive services at the health facility level (the 3^rd^ delay).

**Conclusion:**

The majority of the participants believed preeclampsia as a pregnancy-specific hypertensive disease and mainly associated it with overweight and nutritional problems. The finding of this study implied that awareness creation about the danger of hypertension during pregnancy and its risk reduction mechanisms shall be emphasized. The care provision at health facilities shall be improved by decreasing long waiting time which discourages service utilizations aside from improving early seeking behavior of pregnant women through different interventions.

## Introduction

Preeclampsia is a pregnancy-specific hypertensive disorder characterized by increased blood pressure and excess protein excretion in the urine [1]. It is one of the top maternal morbidity and mortality cause that disproportionately affects pregnant women residing in low and middle-income countries where access and quality of health services are limited [2,3]. One-tenth to one-quarter of maternal death is attributed to hypertensive disorders of pregnancy (HDP) in the developing regions of the world [4,5]. In contrast to other direct maternal mortality causes such as abortion and obstructed labor, mortality related to HDP has an increasing trends [6]. In Ethiopia, an increasing trend in the proportion of preeclampsia was reported by a study conducted in selected governmental hospitals of Addis Ababa. According to the study, the proportion of preeclampsia was increased from 2.2% in 2009 to 5.58 in 2013 [7].

According to studies, HDP particularly preeclampsia is found to be associated with a number of maternal and fetal morbidities [8–10]. Preeclampsia is linked to complications such early birth, fetal growth restriction, intrauterine fetal death, renal or hepatic failure, hemorrhage, and stroke [10,11]. Similarly, a study in Iran found that 10.6%, 7.7%, and 0.3% of women with severe preeclampsia had coagulopathy, placenta abruption, and HELLP syndrome (Hemolysis, Elevated Liver Enzymes, and Low Platelet count), respectively aside from increasing operative deliveries [9]. Another study pointed out that cerebral infarction, congestive heart failure, pulmonary edema, renal failure, and death as complications of preeclampsia [8].

Several risk factors for preeclampsia have been identified. Being of advanced age, primigravida, having a history of preeclampsia, having a short duration of cohabitation, and having a low socioeconomic status are associated with an increased risk of developing preeclampsia. [8,12,13]. Obesity, chronic hypertension, diabetes mellitus, adolescent pregnancy, and conditions that lead to hyper placentation and large placentas are also linked to preeclampsia [4].

However, the perceived cause, severity, and consequences of preeclampsia, as well as perceived barriers to prevention mechanisms, are not well explored, which has a direct or indirect impact on prevention measures, health-seeking behaviors, and management outcomes in women with preeclampsia. People in different areas perceive preeclampsia differently which affects the timing and place of health-seeking. Positive perception about the perceived cause, perceived complications, and prevention of the disease is central for prediction, early diagnosis of the disease which in turn improves maternal and fetal outcomes [14]. While women who had a negative perception towards preeclampsia may experience serious complications due to delay in health-seeking [14]. A study in Nigeria about perception towards eclampsia indicated that nearly sixty percent of the study participants reported that eclampsia is related to the evil spirit and only 6% reported to relate eclampsia as a consequence of increased blood pressure [15]. Similarly, another study conducted in Ogun State, Nigeria detailed that there was a deep-rooted community thought about the cause of preeclampsia/eclampsia which is linked to stress origin [16].

A study in Southern Mozambique disclosed that preeclampsia was not known in the community despite participants were familiar with hypertension and seizure in pregnancy [17]. Similarly, a qualitative study in India revealed that stress, tension, and poor diet were perceived to aggravate hypertension in pregnancy [18]. Perception about preeclampsia, perceived cause, perceived risk, and barriers to early health-seeking behavior are not studied in Ethiopia. In this study, we aimed to assess the perception towards preeclampsia and perceived barriers to early health-seeking among pregnant women in selected Hospitals of South Gondar Zone, Northwest Ethiopia.

## Methods

### Study design, area, and period

A phenomenological study approach was followed to explore the perception of pregnant women towards preeclampsia and perceived barriers to early health seeking. The study was the part of a multicenter study conducted from January to February 2020 in four selected Hospitals (Debre Tabor Hospital, Addis Zemen Hospital, Mechane Eyesus Hospital, and Nefas Mewucha Hospital) found in South Gondar Zone of the Amhara Region [19].

### Study population and sampling technique

Pregnant women who visited the Antenatal Care (ANC) clinics of the selected hospitals were included in the study. Women who had at least one previous birth were purposively selected to better explore their perception regarding preeclampsia, its cause, perceived barriers, and perceived prevention mechanisms. Twenty pregnant women were participated in this study.

### Data collection tool and data collection process

Data were collected through an in-depth interview (IDI) using a semi-structured interview guide (IDI S3). The guide was prepared in the English Language following the review of various literature [14,16,17,20]. Then the guide was translated to the Amharic language which is spoken as a primary language in the study area to ease the communication process. The IDI has focused on perception towards preeclampsia, perceived causes, perceived severity, perceived consequences, perceived prevention methods, and perceived barriers to early health-seeking. The data collection process through IDI was carried out by four BSc Midwives and supervised by four MSc Clinical Midwives. The interview was conducted in a quiet and stable condition to avoid the diversion of attention until information saturation was obtained.

### Data quality assurance

Appropriate recording and abstraction of notes were carried out to maintain the quality of the data. The credibility and dependability of the data were maintained by continuous follow-up aside from triangulation of data in time, person, and place.

### Data analysis

The collected notes were translated into English language and reread by two independent authors. The translated notes were reviewed by all authors for correctness. Following the proofreading of the translated notes, data were organized by coding texts into meaningful elements using open code version 4.03 software. The themes and sub-themes were identified from the data using thematic analysis through the grouping of related codes.

### Operational definition

#### Good health-seeking behavior

Health care-seeking behavior has been defined as any action undertaken by individuals who perceive themselves to have a health problem for finding an appropriate solution [21]. Good health-seeking was considered when the participants prefer to visit health institutions in case of an illness rather than visiting traditional healers.

#### Positive perception

Perception not against the scientific base was considered as positive perception. The perception of pregnant women towards preeclampsia was reported in a descriptive manner [14].

### Ethics approval and consent to participate

The study was approved by the research ethics committee of the College of Health Sciences, Debre Tabor University. A support letter was also obtained from the College following the ethical approval. The study has been performed by following the Helsinki declaration. The purpose of the interview was explained to the IDI participants and written informed consent was taken from each study participant before the commencement of the interview. Permission was obtained from each hospital to conduct the study.

## Results

### Characteristics of the study participants

Twenty pregnant women who visited the selected hospitals for ANC follow-up were included in this study where theoretical information saturation was obtained. Regarding the socio-demographic condition, 12 (60%) and 16 (80%) of the IDI participants were Urban residents and in the age group of 20–34 years respectively. The mean age of the study participants was 29.35 years with a minimum and maximum age of 20 and 38 years respectively. Almost all, 19 (95%) of the study participants were married and 11 (55%) had not attended any formal education. With regards to obstetrics history, 19 (95%) and 8 (40%) of the study participants were gravida 2–4 and para 2–4 respectively (S1 Table).

### Themes of the study

The themes generated from this study were perception towards preeclampsia, perceived cause, perceived severity, perceived consequences, health-seeking behavior, perceived prevention, and perceived barriers to early health-seeking towards preeclampsia (S2 Table).

### Perception about preeclampsia and its risk factors

There was no specific term describing preeclampsia in the study area. The participants described it in English as “high blood pressure in pregnancy/hypertension that occurs at times of pregnancy” in Amharic “*be-ergizina gizie yemikeset yedem gifit*”. Nearly two-thirds of the study participants believed; preeclampsia as a pregnancy-specific hypertensive disease that results in morbidity and mortality on the mother as well as the growing fetus. While the rest one-third did not know anything about preeclampsia. With regards to the risk factors, the study participants had poor awareness about preeclampsia. While some participants believed that lack of physical activity, overweight, malnutrition, being primigravida, and failure to have ANC follow-up are risk factors for preeclampsia (S2 Table).

*“I have experienced this disease in my previous pregnancy*. *It is a hypertension disease that occurs during pregnancy but I don’t know its exact cause*. *It may be associated with overweight”*

*(participant age 30*, *gravida 2 and para 1)*.

*“It is hypertension during pregnancy that leads the mother to death*. *Regarding its cause*, *it is unknown*: *primiparity*, *genetics problems*, *stress*, *and a high altitude might be the cause*"

*(participant age 26*, *gravida 2 and para 1)*.

*"It is a disease of an evil spirit*, *do you know*?, *how hypertension results in edematous of the body and convulsion*?"

*(Participant age 28*, *gravida 3 and para 2)*.

*“Professionals in this hospital told me that it is a hypertension disease that occurs during pregnancy*. *Regarding the cause*, *it may be associated with diabetes mellitus and overweight”*

*(participant age 38*, *gravida 4 and para 2)*.

*“I know this is a disease of obese women*, *it may also be associated with other medical illnesses such as diabetes mellitus*, *renal and cardiac diseases”*

*(participant age 35*, *gravida 3 and para 2)*.

*“I hear something about this disease*. *It is related to nutritional problems and overweight*. *Failing to get treatment for other diseases may also cause this disease”*

*(participant age 28*, *gravida 2 and para 1)*.

### Perceived severity and perceived consequences of preeclampsia

The most of the IDI participants (15 out of 20) believed in the seriousness of preeclampsia both on the health of the mother as well as the fetus. It was believed that leg swelling, severe headache, blurred vision, and epigastric pains as signs of severe preeclampsia by the IDI participants. With regards to the consequences, abortion, preterm birth, convulsion /loss of consciousness of maternal, and fetal death were pointed out as complications of preeclampsia by the IDI participants (S2 Table).

*“Preeclampsia is a dangerous disease which is associated with raised blood pressure*, *severe headache and chest pain”*

*(participant age 26*, *gravida 2 and para 1)*.

*”Dangerous leads to maternal death*. *In the severe form the disease may cause swelling of the leg*, *vision problem and epigastric pain*"

*(participant age 38*, *gravida 4 and para 2)*.

*“I have faced this disease in my previous pregnancy*. *Hence*, *I understand how much dangerous this disease is*. *My pregnancy was terminated before reaching term and I lost my baby*. *God help me to survive this time*. *By the time*, *I decided to go to the hospital and the physician told me that the disease was “hypertension in pregnancy” and for the sake of saving my life*, *the pregnancy was terminated before reaching term”*

*(participant age 31*, *gravida 4 and para 3)*.

### Health seeking behavior and perceived prevention methods

A significant proportion of the IDI participants had good health seeking behavior as they visit health facilities if they encounter preeclampsia. With regards to the perceived prevention of preeclampsia, three-fourth of the IDI participants had agreed that preeclampsia can be prevented through the avoidance of modifiable risky behaviors such as diet adjustments, physical activity, and weight reduction measures. It was also reported that having ANC follow-up would reduce the risk of experiencing preeclampsia (S2 Table).

*“Regarding the prevention*, *early detection of the disease and adjusting nutrition can prevent the disease”*

*(participant age 30*, *gravida 3 and para 1)*.

*“That is why we are here*. *Having ANC follow-up and diet adjustment can help us to prevent this disease”*

*(participant age 38*, *gravida 4 and para 2)*.

*"Since I have faced the disease in my previous pregnancy*, *I want to have a strict follow-up*. *Physical activity*, *eating a balanced diet*, *and avoiding early marriage can help to reduce the risk of this disease”*

*(participant age 31*, *gravida 4 and para 3)*.

*“I never think that having ANC follow-up can reduce the risk associated with this disease*, *rather I come here to have routine ANC”*

*(participant age 27*, *gravida 2 and para 1)*.

*“I don’t know where I have to go if I catch this disease”*

*(participant age 35*, *gravida 3 and para 2)*.

### Perceived barriers to early health-seeking to preeclampsia

Regarding barriers to early health-seeking behavior related to preeclampsia, two themes were developed from the IDI. The themes were self-limited (client-related) barriers and access-related barriers (poor access to health facilities). Within the theme of client-related barriers three subthemes were identified; self-delay, lack of awareness, and low economic status. Within the sub-theme of access-related barriers, three sub-themes were developed; poor infrastructure, long-distance, and poor access to a health facility (S2 Table).

*“Transport problem and poor road access are the barriers to visit health facilities timely related to this disease”*

*(participant age 38*, *gravida 4 and para 2)*.

*”Lack of money*, *transport problem and long-distance compromise early health-seeking related to this disease*"

*(participant age 31*, *gravida 4 and para 3)*.

*“When I want to have ANC follow-up and going to health facilities*, *I am primarily being worried about transport problem and its cost”*

*(participant age 27*, *gravida 2 and para 1)*.

*“Poor awareness related to the disease*, *poor road access*, *and transportation problems affects early health-seeking practice”*

*(participant age 28*, *gravida 2 and para 1)*.

*“The barrier is related to our lack of awareness about the diseases”*

*(participant age 36*, *gravida 4 and para 3)*.

### Perceived barriers to receive care at the health institution level

Under the theme of perceived barriers to receiving care at the health facility, three sub-themes were developed. These were poor time management of professionals, material shortage, and long waiting time. As the study participants reported, there was a long waiting time and poor time management in health institutions which affects the health-seeking behavior of women in case of preeclampsia and other obstetric emergencies (S2 Table).

*“It is very difficult to get service here*, *the health professionals are not serving us as quickly possible; we are forced to wait a long time*"

*(participant*, *age 28*, *gravida 2 and para 1)*.

*“They (the professionals) are wasting time unnecessarily*, *they are not sending us to our home timely by providing proper services which discourage health service utilization”*

*(participant age 32*, *gravida 2 and para 1)*.

## Discussion

A significant proportion of the IDI participants agreed that preeclampsia is a pregnancy-specific hypertension disease that results in complications such as abortion, preterm birth, and convulsion that leads to maternal morbidity and mortality. The finding of our study is supported by studies conducted in Mozambique [17] and Uganda [22]. With regards to its cause, the study participants poorly understood the etiology of preeclampsia. While some tried to associate it with lack of physical activity, weight gain, and poor dietary habits. Similar findings were reported by studies conducted in Nigeria [15] and Uganda [22]. Hence, social behavior change communication on the cause of preeclampsia and its consequences shall be addressed to pregnant women during ANC follow-up to make pregnant women more familiar with the disease and to undertake necessary preventive measures. A study conducted in Nigeria regarding the perception of preeclampsia and eclampsia disclosed that the quality of patient-provider antenatal care interactions, and supportive discussions and care seeking enabling decisions with families and communities [23]. Moreover, enhancing the value of women’s reproductive rights through community-based interventions and increasing health facility-community linkage through the use of community health agents such as women development army, Health Extension workers, and community leaders shall be stressed to improve the health seeking behaviors of pregnant women [24].

Good health-seeking behavior is relevant for the early detection of preeclampsia which reduces complications associated with the disease. In this study, the majority of the IDI participants had good health-seeking behavior towards preeclampsia. A study conducted in Pakistan also reported that women usually visited health facilities when they experienced pregnancy complications or danger signs during pregnancy [25]. In our study, most of the study participants agreed that complications associated with preeclampsia are preventable. Diet adjustment and lifestyle modifications were reported as the main prevention mechanisms of preeclampsia by the study participants. The finding of our study is supported by previous studies [26].

Personal delay, lack of awareness about the disease, transport problem, low socioeconomic condition, and poor access to health facilities were reported as the major barriers to early health-seeking behavior related to preeclampsia. The finding of this study indicated that personal and access-related barriers shall be counteracted to avoid the delay in deciding to seek care (1^st^delay) and the delay to reach the health facility (2^nd^ delay) respectively to reduce morbidity and mortality associated with preeclampsia. A similar finding was reported in previous studies conducted in, Uganda [27], Nigeria [15], and Pakistan [25]. According to studies conducted in different countries, context-based community awareness, improving access, and availability of health services are critical in promoting health-seeking behavior and the quality of health services [23,26,28] which also can be implemented in Ethiopian context. Delay in the management of preeclampsia might lead to eclampsia, resulting in placental insufficiency and organ dysfunction [4]. Ensuring equitable services and establishing emergency supportive care in rural areas to care for pregnant and laboring women with preeclampsia shall be strengthened to reduce morbidity and mortality associated with preeclampsia [3]. Similarly, establishing preventive and treatment centers to the nearest possible where pregnant women live could be a substantial means to reduce the burden attributable to preeclampsia [29].

In this study, not receiving proper care and long waiting time were the main barriers to receive care at the health facility level as experienced by the study participants (the 3^rd^ delay). Delay in providing proper care might cause serious maternal and perinatal morbidity and mortality [30]. Hence, improving the quality of the care provisions and reducing long waiting times are essential measures to improve the health-seeking behavior and treatment outcome of preeclampsia [31]. Stakeholders shall strengthen the health care delivery mechanisms of hospitals to increase the community’s trust in the health system [32].

## Strength and limitation of the study

The perception of pregnant women towards preeclampsia was explored qualitatively in this study which can be taken as strength of the study. While this study shall be inferred in the consideration of the limitation that women who had ANC follow-up might be different from pregnant women who had no ANC follow-up.

## Conclusion

The majority of the IDI participants believed preeclampsia as a pregnancy-specific hypertensive disease and mainly associated it with overweight and nutritional problems. With regards to the perceived severity, the IDI participants agreed that preeclampsia can lead women to death. Personal delay, lack of awareness about the disease, transport problem, and low socioeconomic condition were perceived as the major reasons for the delay to early health-seeking (the 1^st^ and the 2^nd^ delay). While poor service provision and long waiting times were the barriers to receive services at the health facility level (the 3^rd^ delay). The finding of this study implies that awareness creation about the danger of hypertension during pregnancy and its risk reduction mechanisms shall be emphasized. The care provision at health facilities shall be improved since there is a long waiting time which discourages service utilizations aside from improving early health seeking behavior.

## Supporting information

S1 Table Sociodemographic and obstetrics characteristics of the study participants. (DOCX)

S2 Table Themes and main findings explored about perception towards preeclampsia. (DOCX)

S1 File IDI S3: In-depth interview guide perception towards preeclampsia. (DOCX)

## Abbreviations

ANC: Antenatal Care
HDP: Hypertensive Disorders of Pregnancy
HELLP: syndrome: Hemolysis, Elevated Liver Enzymes, and Low Platelet count
IDI: In-depth Interview
