# Supplementary material for: Perception towards preeclampsia and perceived barriers to early health-seeking among pregnant women in selected Hospitals of South Gondar Zone, Northwest Ethiopia: A qualitative study
Source: PLoS One. 2022 Aug 4;17(8):e0271502. doi: 10.1371/journal.pone.0271502 (PMC9352094; doi:10.1371/journal.pone.0271502)
Supplement: S1 Table — (DOCX) [file pone.0271502.s001.docx]

Table S1: Sociodemographic and obstetrics characteristics of the study participants

| **Variables** | **Frequency (n)** | **Percent (%)** |
| --- | --- | --- |
| **Residence** | | |
| Urban | 12 | 60% |
| Rural | 8 | 40% |
| **Age** | | |
| 20-34 years | 16 | 80% |
| ≥ 35 years | 4 | 20% |
| Mean age | 29.35 years | |
| **Marital status** | | |
| Married | 19 | 95% |
| Widowed | 1 | 5% |
| **Education status** | | |
| No formal education | 11 | 55% |
| Primary school | 7 | 35% |
| Secondary school | 2 | 10% |
| **Gravida** |  |  |
| 2-4 | 19 | 95% |
| ≥ 5 | 1 | 5% |
| **Para** | | |
| 1 | 12 | 60% |
| 2-4 | 8 | 40% |
