# Supplementary material for: Perception towards preeclampsia and perceived barriers to early health-seeking among pregnant women in selected Hospitals of South Gondar Zone, Northwest Ethiopia: A qualitative study
Source: PLoS One. 2022 Aug 4;17(8):e0271502. doi: 10.1371/journal.pone.0271502 (PMC9352094; doi:10.1371/journal.pone.0271502)
Supplement: S2 Table — (DOCX) [file pone.0271502.s002.docx]

Table S2:Themes and main findings explored about perception towards preeclampsia

| *Themes* | *Narrations /main findings/ from participates* |
| --- | --- |
| *Perception about preeclampsia and its risk factors* | *“I have experienced this disease in my previous pregnancy. It is a hypertension disease that occurs during pregnancy but I don’t know its exact cause. It may be associated with overweight” (P7Age 30G2P1)*  *“Very dangerous disease, leading the mother to death. Regarding its cause, it is unknown: primi-parity, genetics problems, stress and high altitude might be the cause” P9 Age26G2P1*  *“It is a disease of evil spirit, do you know how hypertension results edematous of the body and convulsion?” P13Age 28G3P2.*  *“Professionals in this hospital told me that It is a hypertension disease that occur during pregnancy. Regarding the cause it may be associated with diabetes mellitus and overweight” P14Age38G4P2.*  *I know this is a disease of obese women, it may also associated with other illnesses such as diabetes mellitus, renal and cardiac diseases”” P16Age35G3P2* |
| *Perceived severity of the disease* | *“Dangerous disease associated with raised blood pressure, severe headache and chest pain” P9 Age26G2P1.*  *”Dangerous leads to maternal death” P13Age 28G3P2.”In severe form the disease may cause swelling of leg, vision problem and epigastric pain” P14 Age38G4P2.*  *“I have faced this disease in my previous pregnancy. Hence, I understand how much dangerous this disease is. My pregnancy was terminated before reaching term and I lost my baby. God help me to survive this time. by the time, I decided to go to hospital and the physician told me that the disease was preeclampsia and for the sake of saving my life, the pregnancy was terminated before reaching term” P12 Age31G4P3.* |
| *Perceived consequence* | *It can results “preterm birth, abortion, and death. Generally, it wastes pregnancy time of the mother” P11Age26G2P1.* |
| *Health seeking, prevention methods* | *“Early detection of the disease and adjusting nutrition can prevent the disease” P7Age30G2P1.*  *“Since I have faced the disease in my previous pregnancy, I want to have strict follow-up. Physical activity, eating balanced diet and avoiding early marriage can help to reduce the risk of this disease” P12Age31G4P3.*  *“That is why we are here. Having ANC follow-up and diet adjustment can prevent this disease” P14 Age38G4P2.*  *“I never think that having ANC follow-up can reduce the risk associated with this disease, rather I come here to have routine ANC” P17 Age27G2P1.* |
| *Barriers to early health seeking for preeclampsia* | *”shortage of money, transport problem and long distance compromise early health seeking related to this disease” P12 Age31G4P3.*  *Poor awareness related the disease, poor road access, and transportation problems” affects early health seeking practice P8 Age26G2P1.*  *“When we want to have ANC follow-up and going to health facilities we are being worried about transport problem and its cost” P17 Age27G2P1.*  *“Transport problem and poor road access are the barriers to visit health facilities timely related to this disease” P14 Age38G4P2.* |
| *Barriers to receive care at health facility* | *“It is very difficult to get service here, they are not serving us as quickly possible; women are forced to wait long time” P18 Age28G2P1.*  *“They (the professionals) are wasting time unnecessarily, they are not sending us to our home timely by providing proper services which discourages health service utilization ” P10 Age32G2P1* |
