# Supplementary material for: Perception towards preeclampsia and perceived barriers to early health-seeking among pregnant women in selected Hospitals of South Gondar Zone, Northwest Ethiopia: A qualitative study
Source: PLoS One. 2022 Aug 4;17(8):e0271502. doi: 10.1371/journal.pone.0271502 (PMC9352094; doi:10.1371/journal.pone.0271502)
Supplement: S1 File — (DOCX) [file pone.0271502.s003.docx]

**English version Indepth interview (IDI) guide**

The in-depth interview will be addressed based on perceived cause, perceived consequences and perceived prevention methods.

**Age of the participants-------- gravida-------para-------**

**Marital status ------------- educational level -------------- Residence ______________**

**In your opinion, what do you understand about preeclampsia /pregnancy induced hypertension? (**Probing will be done by using (terms family related issues, nutrition)

- 1. What is your perception about preeclampsia
  2. Your perception about severity of the disease

----------------------------------------------------------------------------------------------------

- 1. Your perceptions about its cause , risk factors, clinical features

---------------------------------------------------------------------------------------------------

- 1. Your health care seeking behavior for preeclampsia

------------------------------------------------------------------------------------------------

1. **What is the preceived consequencs of preeclampsia?**

(prob, Convulsion, preterm birth, fetal death, maternal death etc )

-------------------------------------------------------------------------------------------

1. **In your opinion what is prerceived prevention methods of preeclampsia?**

(probe: nutrition, early detection, delay pregnancy, cultural etc)

---------------------------------------------------------------------------------------------

1. Have you ever visited any health facility for preeclampsia before?----------------------
2. In your opinion , what socio-cultural, geographical, economical barriers do you think affect women to seek early medical care for hypertension in pregnancy? -----------------------------------------------------------------------------------------------------------------------------------
3. In your opinion, what barriers did you observe in the health facilities for seeking care for preeclampsia? (Prob. Challenges in the hospital related issues like availblity of services and health care professionals, diagnostic and other fasility related issues).
4. Do you have any additional infromation you want to add which is not mentioned before ?--------------------------------------------------------------------------------------------------------------------

**Thank you for your cooperations !!!**

**Amharic version Indepth Interview Guide (የአማርኛ ቃለ መጠይቅ መመሪያ)**

**የተሳታፊ ዕድሜ -------- የእርግዝን ብዛት (ግራቪዳ)-------የወሊድ ብዛት (ፓራ) ------**

**የጋብቻ ሁኔታ ------------- የተሳታፊዎች የትምህርት ደረጃ --------- የመኖሪያ ቦታ_________________**

1. በእርስዎ አስተያየት ፣ ፕሪኤክላምፕሲያ / በእርግዝና ምክንያት ስላለው የደም ግፊት ምን ተረድተዋል? (ምርመራ ማድረግ (ከቤተሰብ ጋር የተያያዙ ጉዳዮች ፣ አመጋገብ እና የመሳሰሉት)

1.1. ስለ ፕሪኤክላምፕሲያ ያለዎት ግንዛቤ ምንድን ነው?

1.2. ስለ በሽታው ከባድነት ያለዎት ግንዛቤ

----------------------------------------------------- -----------------------------------------------------

1.3. ስለ መንስኤው ፣ ለአደጋ ተጋላጭ ምክንያቶች ፣ ስለ በሽታው ገጽታዎች ያለዎት ግንዛቤ እንዴት ነው?

----------------------------------------------------- -------------------------------------------------

1.4. ፕሪኤክላምፕሲያ የጤና እንክብካቤ ፍለጋ ባህሪዎ እንዴት ነው?

----------------------------------------------------- ---------------------------------------------

2. የፕሪኤክላምፕሲያ ቅድመ መዘዝ ምንድነው?

(**ምርመራ** : ራስን መሳት ፣ ያለጊዜው መወለድ ፣ የፅንስ ሞት ፣ የእናቶች ሞት ወዘተ)

----------------------------------------------------- ---------------------------------------------

3. በእርስዎ አስተያየት ፕሪግላምፕሲያ ቅድመ መከላከያ ዘዴዎች ምንድናቸው?

(**ምርመራ-**አመጋገብ ፣ ቅድመ ምርመራ ፣ መዘግየት እርግዝና ፣ ባህላዊ ወዘተ)

----------------------------------------------------- ---------------------------------------------

4. ከዚህ በፊት ለፕሪኤክላምፕሲያ ማንኛውንም የጤና ተቋም ጎብኝተው ያውቃሉ? ----------------------

5. በአስተያየትዎ በእርግዝና ወቅት ለደም ግፊት ከፍተኛ የሕክምና እርዳታ ለመፈለግ በሴቶች ላይ ምን ተጽዕኖ ያሳድራል? (**ምርመራ:** ማኅበራዊ ፣ ባህላዊ ፣ ጂኦግራፊያዊ ፣ ኢኮኖሚያዊ እንቅፋቶች ወዘተ)? ----------------------------------------------------- ----------------------------------------------------- -----------------------------------

6. በአስተያየትዎ የፕራይፕላምፕሲያ እንክብካቤን ለመፈለግ በጤና ተቋማት ውስጥ ምን መሰናክሎችን ታዘቡ? (እንደ ሆስፒታል አገልግሎት እና የጤና እንክብካቤ ባለሙያዎች ፣ የምርመራ እና ሌሎች ፋሲሊቲ ጉዳዮች ያሉ ሆስፒታሎች ጋር ተያያዥነት ያላቸው ችግሮች )

7. ከዚህ በፊት ያልተጠቀሰው ሊያክሉት የሚፈልጉት ተጨማሪ ሃሳብ ይኖርዎታል? -------------------------------- -----

**ስለ ትብብርዎ አመሰግናለሁ!!!**
